# Guidelines to Aid in the Distinction of Endometrial and Endocervical Carcinomas, and the Distinction of Independent Primary Carcinomas of the Endometrium and Adnexa From Metastatic Spread Between These and Other Sites

**DOI:** 10.1097/PGP.0000000000000553

**Published:** 2018-12-14

**Authors:** Colin J.R. Stewart, Christopher P. Crum, W. Glenn McCluggage, Kay J. Park, Joanne K. Rutgers, Esther Oliva, Anais Malpica, Vinita Parkash, Xavier Matias-Guiu, Brigitte M. Ronnett

**Affiliations:** Department of Histopathology, King Edward Memorial Hospital and School for Women’s and Infants’ Health, University of Western Australia, Perth, Western Australia, Australia (C.J.R.S.); Department of Pathology, Brigham and Women’s Hospital (C.P.C.); Department of Pathology, Massachusetts General Hospital and Harvard Medical School (E.O.), Boston, Massachusetts; Department of Pathology, Belfast Health and Social Care Trust, Belfast, Northern Ireland, UK (W.G.M.); Department of Pathology, Memorial-Sloan Kettering Cancer Center, New York, New York (K.J.P.); Department of Pathology and Laboratory Medicine, Cedars-Sinai Medical Center, Los Angeles, California (J.K.R.); Department of Pathology, The University of Texas MD Anderson Cancer Center, Houston, Texas (A.M.); Department of Pathology, Yale University School of Medicine, New Haven, Connecticut (V.P.); Pathological Oncology Group and Pathology Department, Hospital Arnau de Vilanova, Lleida, Spain (X.M.-G.); Departments of Pathology and Gynecology and Obstetrics, The Johns Hopkins University School of Medicine, Baltimore, Maryland (B.M.R.)

**Keywords:** Endometrial, Endocervical, Ovary, Carcinoma, Distinction, Metastasis, Independent, Synchronous

## Abstract

In most cases of suspected endometrial neoplasia tumor origin can be correctly assigned according to a combination of clinical, radiologic, and pathologic features, even when the latter are based upon the examination of relatively small biopsy samples. However there are well-recognized exceptions to this rule which continue to create diagnostic difficulty, and sometimes difficulties persist even after the detailed examination of resection specimens. Among the most common problems encountered in practice are the distinction of primary endometrial and primary endocervical adenocarcinomas, and the determination of tumor origin when there is synchronous, multifocal involvement of gynecologic tract sites, for example the endometrium and the ovary. However, accurate diagnosis in these cases is important because this has significant staging, management and prognostic implications. In this review we discuss the value and limitations of key morphologic, immunophenotypic and molecular findings in these diagnostic scenarios.

Endometrial carcinoma is the most common gynecologic malignancy in most developed countries and usually the diagnosis is suspected based upon clinical and imaging findings and confirmed with endometrial biopsy. In some cases it can be difficult to determine whether a tumor (usually an adenocarcinoma) has arisen within the uterine corpus or the cervix but this distinction is important because the optimal management of endometrial and cervical carcinoma differs significantly. Similar difficulty can occur when there is multifocal tumor distribution, for example synchronous neoplasia involving the endometrium, fallopian tube and/or ovary, and tumor origin may remain uncertain even after the detailed examination of surgically resected hysterectomy and salpingo-oophorectomy specimens. The ultimate interpretation of such cases as representing either multifocal low-stage neoplasia or metastasis from one anatomic site to another is critical since this affects tumor staging and management including the recommendation for adjuvant chemotherapy and/or radiotherapy. While ancillary studies such as immunohistochemistry are often helpful in these problematic cases, it is emphasized that the selected antibody panel must be tailored according to the specific diagnostic context, otherwise the results can be unhelpful or even misleading. Molecular analysis is likely to be increasingly applied in this setting and may provide insights into tumor clonality as well as identifying potential avenues for targeted therapy. However, the interpretation of molecular findings is not always straightforward and these tests are often not readily available.

In this review we discuss the value and the limitations of morphologic, immunohistochemical, and molecular findings in cases where there is uncertainty regarding tumor origin. While this review will focus upon primary gynecologic neoplasia, potential problems presented by tumors metastatic to the endometrium will also be briefly discussed.

## DISTINCTION OF ENDOCERVICAL AND ENDOMETRIAL ADENOCARCINOMA

The distinction between an endocervical and an endometrial origin for an adenocarcinoma is important for optimal patient management. There are a number of reasons why distinction of these adenocarcinomas can be problematic. These include overlap in morphology and involvement of both sites by tumor on imaging studies as well as on biopsy specimens and on gross and microscopic evaluation of a hysterectomy specimen. Moreover, the dominant tumor component in a hysterectomy specimen may not represent the primary site, as spread of a smaller primary tumor can result in a larger tumor at another site. While immunohistochemical analysis is often helpful in determining the site of tumor origin, the most valuable panel of markers depends upon the specific subtypes of endometrial and endocervical adenocarcinomas being considered in the differential diagnosis, and it stressed that a standard panel of markers cannot help in distinguishing between all endometrial and endocervical adenocarcinomas. In particular, the choice of markers depends on whether the endometrial adenocarcinoma is endometrioid or serous in type, whether the endometrioid adenocarcinoma is low-grade or high-grade, and whether the cervical neoplasm is a high-risk human papillomavirus (HPV)-related (usual type) adenocarcinoma or one of the less common subtypes unrelated to HPV (e.g. gastric-type, mesonephric or clear cell adenocarcinoma). While it is impossible to cover every possible diagnostic scenario, the most commonly encountered are discussed below.

### Distinction Between High-risk HPV-related (Usual Type) Endocervical Adenocarcinoma and Low-grade Endometrial Endometrioid Adenocarcinoma

**FIG. 1:**
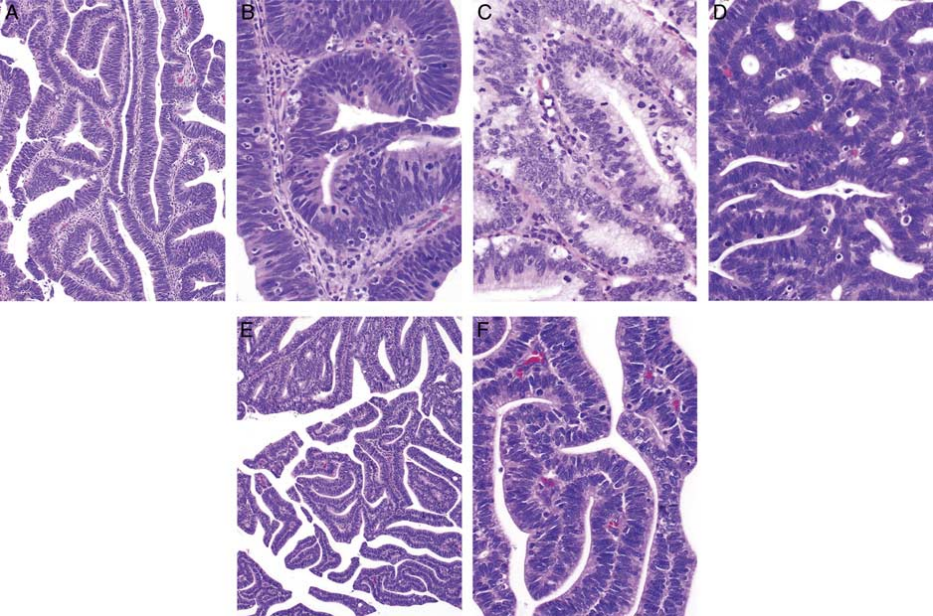
High-risk human papillomavirus–related endocervical adenocarcinoma. Typical example displays a hybrid of endometrioid and mucinous features, with numerous mitotic figures and apoptotic bodies (A, B). Some tumors have more mucinous differentiation (C) or pseudoendometrioid appearances (D). A villoglandular architecture may be seen, similar to the villoglandular variant of endometrial endometrioid carcinoma, but characteristic cytologic features (hybrid endometrioid and mucinous differentiation, numerous mitotic figures and apoptotic bodies) are useful for recognition as a high-risk human papillomavirus–related endocervical adenocarcinoma (E, F).

Difficulties in the distinction between a high-risk HPV-related (usual type) endocervical adenocarcinoma and the common endometrial endometrioid adenocarcinoma (including subtypes of the latter which are usually low-grade), are related to the following:

1. Shared cellular differentiation. Both tumor types can have mucinous and endometrioid-like features. High-risk HPV-related endocervical adenocarcinomas are most often characterized by a hybrid of mucinous and endometrioid-like features, often with numerous apically situated (suspended) mitotic figures and basally situated apoptotic bodies (Figs. 1A, B). Nuclear hyperchromasia is common, as is a pattern of “punched-out” lumina. Varying degrees of mucinous differentiation are often present, with occasional cases dominated by mucinous features, although this is relatively uncommon (Fig. 1C). Some cases display endometrioid-like features (pseudoendometrioid pattern) but true endometrioid differentiation is uncommon (Fig. 1D); in particular, the bland squamous elements (morules) characteristic of many low-grade endometrioid adenocarcinomas are rarely seen in endocervical adenocarcinoma. Most endometrial adenocarcinomas are of the endometrioid type but can have varying degrees of mucinous differentiation and the designation of mucinous adenocarcinoma is made when >50% of the tumor cells contain abundant intracytoplasmic mucin. Low-grade endometrioid adenocarcinomas usually do not have the notable mitotic activity and the numerous apoptotic bodies that characterize high-risk HPV-related endocervical adenocarcinomas but exceptions can occur.
2. Shared architecture. Tumors of both types most often show predominant glandular architecture and both can have papillary and villoglandular growth patterns (Figs. 1E, F).
3. Involvement of both the endometrium and endocervix in biopsy/curettage or hysterectomy specimens.
4. Lack of an identifiable precursor lesion. This is often a problem in biopsy and curettage specimens but can also be problematic in hysterectomy specimens when tumors involve both the endometrium and the endocervix (often including the intervening lower uterine segment). In this situation, precursor lesions can be either overgrown by carcinoma or a precursor lesion may be simulated (e.g. endocervical adenocarcinoma extending into the endometrium and simulating atypical hyperplasia 1, or endometrial endometrioid adenocarcinoma extending into the endocervix and mimicking a premalignant or malignant endocervical glandular proliferation).
5. The dominant tumor component does not necessarily represent the primary site. Some endocervical adenocarcinomas can have limited endocervical involvement and dominant endometrial or endomyometrial involvement, simulating a primary endometrial adenocarcinoma with endocervical extension 1. Conversely, small endometrial adenocarcinomas can exhibit extensive cervical involvement.

**FIG. 2:**
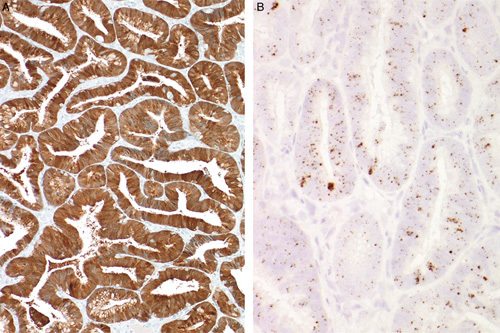
High-risk human papillomavirus–related endocervical adenocarcinomas are characterized by diffuse p16 expression (A) related to the presence of high-risk human papillomavirus which can be detected by DNA *in situ* hybridization (B).

**FIG. 3:**
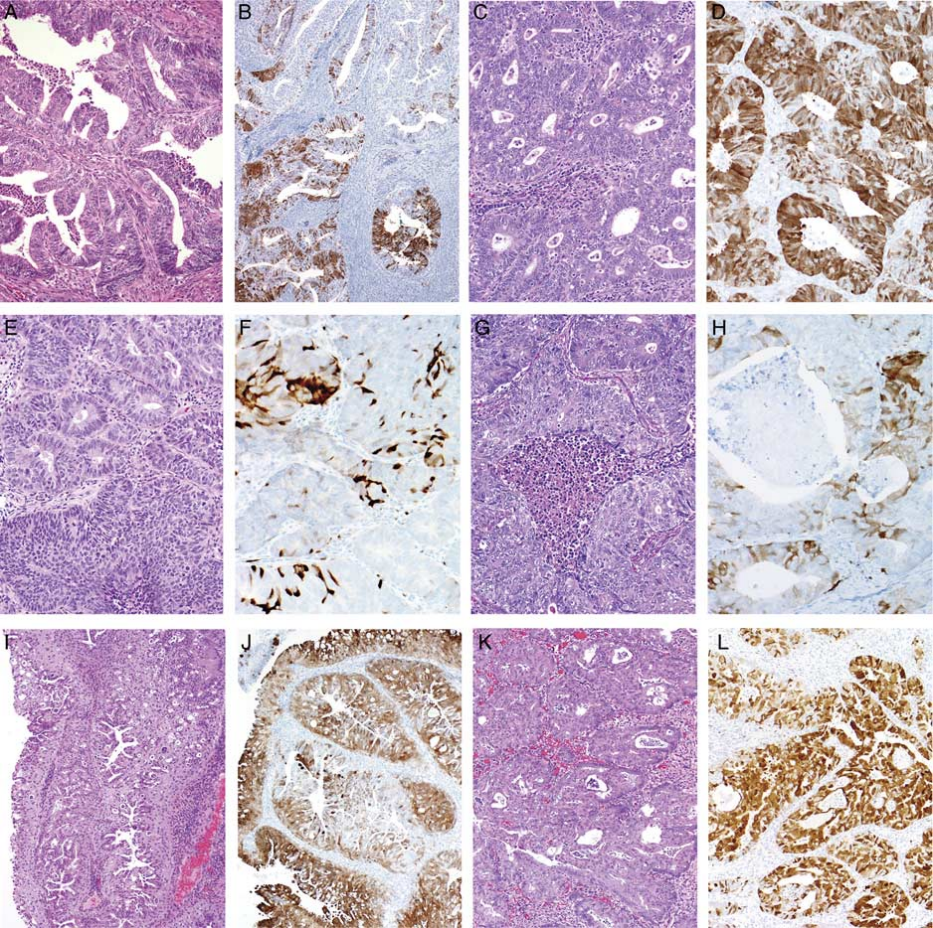
Endometrial endometrioid adenocarcinomas. Typical low-grade (FIGO grade 1) tumor (A) has patchy expression of p16 (B). Some tumors, regardless of grade (another FIGO grade 1 example, C) can have more extensive p16 expression but there are usually interspersed negative foci (D) so that the pattern is not fully diffuse as in a high-risk human papillomavirus–related endocervical adenocarcinoma (compare with Fig. 2A). Most higher-grade tumors (FIGO grade 2 and 3 examples) also have patchy p16 expression (E–H). Some low-grade tumors with extensive metaplastic-type differentation can have more extensive p16 expression but typically there are interspersed negative foci (I–L).

In routine practice, a few selected immunohistochemical markers can readily distinguish most cases of low-grade endometrial endometrioid adenocarcinoma and high-risk HPV-related endocervical adenocarcinoma (Table 1). Carcinoembryonic antigen (CEA) and vimentin have traditionally been used for this distinction but the most useful immunohistochemical markers currently recommended are p16 and hormone receptors [estrogen and progesterone receptor (ER/PR)] 2–5. Use of a panel of markers, rather than a single marker, is encouraged because any immunohistochemical analysis can produce unexpected positive and negative (aberrant) staining reactions with an individual marker but generally not with a range of markers. p16 is probably the single most useful marker for distinction of high-risk HPV-related endocervical adenocarcinomas from endometrial endometrioid adenocarcinoma, but as discussed below and illustrated in Figures 2 and 3 the pattern of staining is critical to interpretation. High-risk HPV-related endocervical adenocarcinomas exhibit diffuse moderate to strong p16 expression (essentially all tumor cells are positive, so-called “block positivity”) which is related to HPV-mediated molecular alterations that result in p16 overexpression (Fig. 2A). Thus, diffuse moderate to strong p16 staining serves as a surrogate marker for high-risk HPV in this setting. High-risk HPV detection within tumor tissue by *in situ* hybridization or other molecular methods is definitive for identification of this group of endocervical adenocarcinomas (Fig. 2B); however, DNA *in situ* hybridization assays and other molecular methods are not 100% sensitive and are not always readily available. Hormone receptors (ER/PR) are also useful but are less discriminatory by themselves

**TABLE 1:**
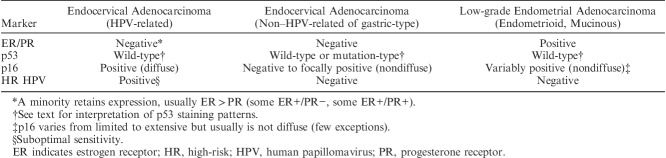
Biomarkers for distinguishing endocervical and low-grade endometrial adenocarcinomas

Endometrial endometrioid adenocarcinomas of all grades almost always display variable patchy p16 expression ranging from limited to extensive, with the more positive tumors still usually having <80% of the cells positive (nondiffuse or mosaic pattern), with scattered negative foci or interspersed individual negative tumor cells present (Figs. 3A–H). Some endometrioid adenocarcinomas, including those with prominent mucinous/metaplastic-type differentiation can exhibit more extensive p16 expression but the staining intensity is usually not as strong as that seen in high-risk HPV-related endocervical adenocarcinomas, and some negative patches are usually present if the sample is not too small (unpublished observations) (Figs. 3I–L). A very small subset of endometrial endometrioid adenocarcinomas exhibit diffuse strong (block type) p16 expression, similar to that seen in HPV-related cervical adenocarcinomas.

**FIG. 4:**
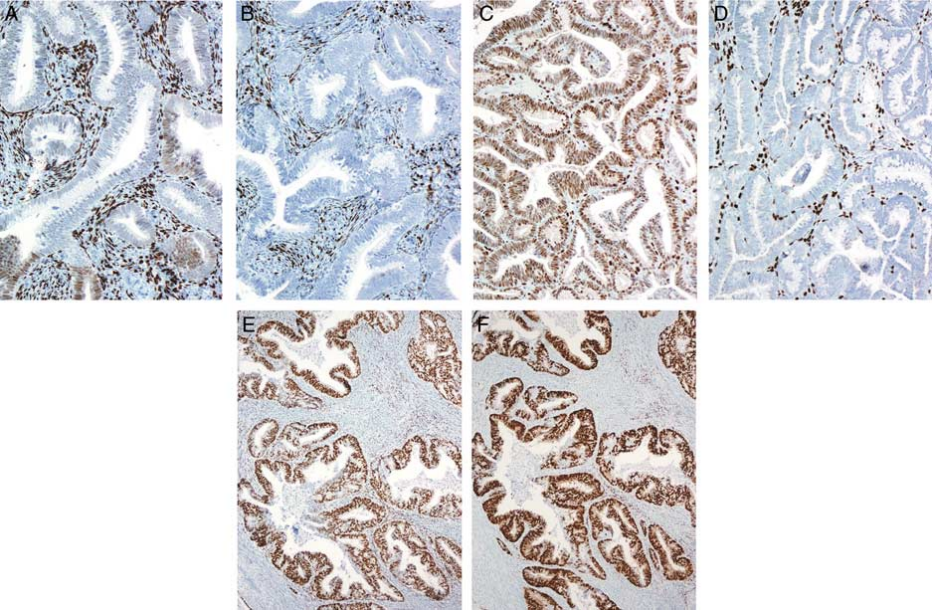
High-risk human papillomavirus–related endocervical adenocarcinomas most commonly are negative with hormone receptors [both estrogen receptor (ER) and progesterone receptor (PR)] (A, B) but some can retain expression to some degree, often ER (C) without PR (D). In contrast, endometrial endometrioid adenocarcinomas usually (but not always) express ER (E) and PR (F).

High-risk HPV-related endocervical adenocarcinomas are typically negative for ER/PR expression (Figs. 4A, B). Some tumors have retained ER expression (albeit typically weaker and focal compared with the usual strong expression in normal glands) with loss of PR expression (Figs. 4C, D), and some retain significant expression of both ER and PR; thus, PR is more discriminatory than ER (unpublished observations). Endometrial endometrioid adenocarcinomas typically express both ER and PR (Figs. 4E, F) but some tumors, particularly but not exclusively high-grade carcinomas, can be negative 6. Thus, p16 immunohistochemistry alone or in combination with ER/PR, distinguishes HPV-related endocervical adenocarcinoma from low-grade endometrioid adenocarcinoma in most cases.

Some, but not all, pathologists find CEA and vimentin to be of some value in the distinction of high-risk HPV-related endocervical adenocarcinomas from endometrial endometrioid adenocarcinomas 7–9. Most of the latter are vimentin positive and CEA negative, whereas most HPV-related endocervical adenocarcinomas show an inverse staining pattern. However, in practice use of these markers is problematic for several reasons: (1) expression can be focal in some cases; (2) CEA staining can be difficult to interpret because squamous elements (commonly seen in endometrioid adenocarcinomas) can be positive, apical/glycocalyceal staining may be seen in endometrioid adenocarcinomas, and some usual type endocervical adenocarcinomas are CEA negative; (3) it may be difficult to ascertain whether vimentin expression is actually within glands versus closely apposed stromal cells; and (4) tumors with mucinous differentiation sometimes express CEA and may be vimentin negative, regardless of origin. Of note, CD10 has no role in determining tumor location or origin as CD10 expression is not specific for endometrial stroma (endocervical stroma can also be positive) 10.

### Distinction Between High-risk HPV-related (Usual Type) Endocervical Adenocarcinoma and High-grade Endometrial Adenocarcinoma

**FIG. 5:**
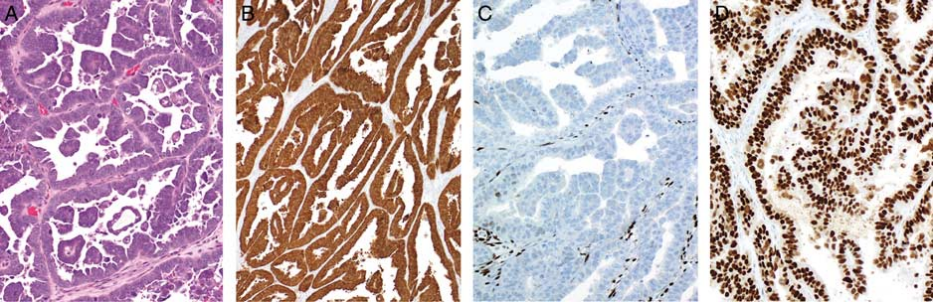
Endometrial serous adenocarcinoma. A typical example is characterized by well formed glandular and papillary structures with high-grade nuclear atypia (A). Characteristically, tumors exhibit diffuse p16 expression (B), are sometimes negative with hormone receptors (C) (estrogen receptor shown; progesterone receptor was also lost), and aberrant p53 expression (D, diffuse over-expression pattern).

The aforementioned panel of markers has some value for distinguishing high-risk HPV-related endocervical adenocarcinomas from high-grade endometrial endometrioid adenocarcinomas (Table 2). However, this panel has significant limitations for distinguishing between an endometrial and a cervical origin for other high-grade carcinomas, including endometrial serous adenocarcinoma and undifferentiated carcinoma 11. While the morphologic features usually differ significantly from high-risk HPV-related endocervical adenocarcinomas, there can be overlap. For example, endocervical adenocarcinomas and endometrial serous adenocarcinoma can both have a papillary and glandular architecture and the nuclear features in each are commonly high-grade. Endometrial serous adenocarcinomas, and some undifferentiated endometrial carcinomas, are characterized by diffuse/strong p16 expression (likely due to non–HPV-related molecular mechanisms affecting the pRb pathway) (Figs. 5A, B). Hormone receptor expression is commonly negative or only focally positive in both of these tumors (Fig. 5C) although some endometrial serous adenocarcinomas show diffuse staining, especially with ER. Thus, if p16 and hormone receptor immunohistochemistry is used in an attempt to distinguish between endometrial and cervical tumor origin, a pattern of diffuse p16 expression and negative or focal hormone receptor expression could result in the misdiagnosis of a primary cervical adenocarcinoma, with resultant inappropriate management.

**TABLE 2:**
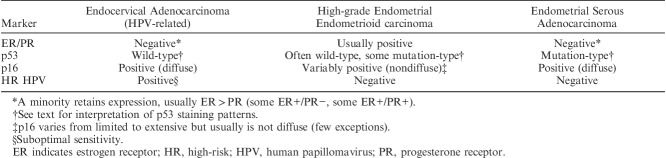
Biomarkers for distinguishing endocervical and high-grade endometrial adenocarcinomas

In this scenario, immunohistochemical analysis of p53 expression and HPV studies may be of value although, as already discussed, the latter may not be readily available. Most endometrial serous adenocarcinomas exhibit one of the 3 patterns of aberrant or “mutation-type” p53 staining because they are associated with *TP53* mutations 12–14. These patterns are described in detail in another review in this series (Interpretation of p53 Immunohistochemistry in Endometrial Carcinomas: Towards Increased Reproducibility), and include the most common diffuse strong positive nuclear reaction (overexpression pattern) (Fig. 5D), the less common complete absence of nuclear expression pattern (null-pattern), and the very uncommon cytoplasmic pattern of immunoreactivity. Although most endometrial serous adenocarcinomas and some undifferentiated carcinomas show diffuse p16 expression, this is not related to high-risk HPV. Because HPV-related molecular alterations, high-risk HPV-related endocervical adenocarcinomas rarely, if ever, harbor *TP53* mutations and thus lack the aberrant (mutation-type) p53 expression patterns seen in serous adenocarcinomas and some other high-grade endometrial carcinomas; rather, high-risk HPV-related endocervical adenocarcinomas almost always exhibit a heterogenous (wild-type) pattern of p53 expression, characterized by scattered weak to moderate nuclear expression in some tumor cells. There remains some controversy as to whether high-risk HPV and *TP53* mutations can coexist within the same neoplasm given that aberrant (mutation-type) p53 staining is rarely seen in high-risk HPV-positive tumors. Therefore in practice the presence of aberrant (mutation-type) p53 staining in a tumor thought to represent a high-risk HPV-related usual type endocervical adenocarcinoma mandates exclusion of a high-grade endometrial or upper genital tract (fallopian tube/ovary) primary. However, it is important to remember that there is a subset of primary cervical adenocarcinomas that are unrelated to high-risk HPV (discussed below), and these tumors may exhibit mutation-type p53 expression. p63 may also assist in certain circumstances in that diffuse nuclear staining in a diffusely p16 positive high-grade carcinoma suggests a poorly differentiated primary cervical squamous cell carcinoma, although squamous elements in endometrioid adenocarcinomas can be p63-positive 15.

Theoretically, a serous adenocarcinoma may arise as a primary cervical neoplasm. However, we believe this to be extremely uncommon and many tumors reported as primary cervical serous adenocarcinomas likely represent usual high-risk HPV-related cervical adenocarcinomas with a “serous-like” morphology or misclassified primary endometrial or even upper genital tract serous adenocarcinomas secondarily involving the cervix.

### Non–HPV-related Endocervical Adenocarcinomas

**FIG. 6:**
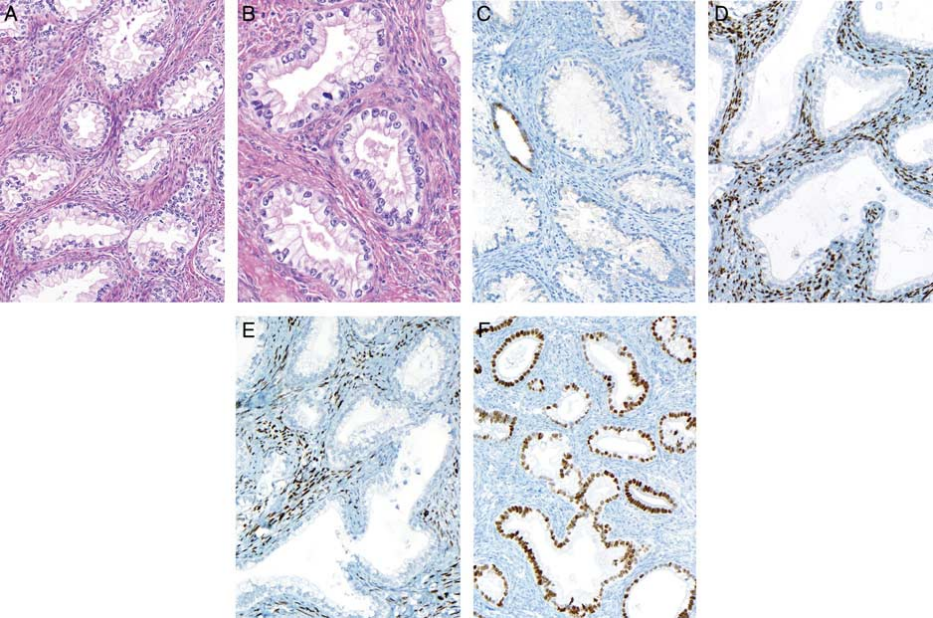
Gastric-type cervical mucinous adenocarcinoma. Typical example is composed of well formed mucinous glands with discrete cell borders (A, B). Most tumors are negative or focally positive for p16 (C) and are negative with hormone receptors estrogen receptor (D) and progesterone receptor (E). Some exhibit aberrant/mutation-type p53 expression (F).

A variety of cervical adenocarcinomas of unusual morphologic subtypes are unrelated to high-risk HPV. These include gastric-type adenocarcinomas (Figs. 6A, B), most clear cell carcinomas, and mesonephric adenocarcinomas. In distinguishing these tumor types from a primary endometrial adenocarcinoma, different panels of markers need to be employed which depend on the subtype of the cervical and endometrial adenocarcinoma being considered. These non–HPV-related cervical adenocarcinomas are almost always p16-negative or focally positive (although rare cases are diffusely positive due to non–HPV-related mechanisms) (Fig. 6C) 16–18. Gastric-type cervical adenocarcinomas are mucinous adenocarcinomas and include the highly differentiated form referred to as adenoma malignum (mucinous variant of minimal deviation adenocarcinoma). Gastric-type adenocarcinomas share negative hormone receptor expression with high-risk HPV-related endocervical adenocarcinomas (Figs. 6D, E). A minority [41% in the only large study to investigate this 19] display aberrant (mutation-type) p53 expression (Fig. 6F) 19. In contrast, as noted previously, high-risk HPV-related (usual type) endocervical adenocarcinomas exhibit heterogenous (wild-type) p53 expression. Confusion may arise between a gastric-type cervical adenocarcinoma and an endometrial mucinous adenocarcinoma (endometrioid carcinoma with extensive mucinous differentiation). Assessment of hormone receptor expression is most useful for this distinction, as the gastric-type cervical adenocarcinomas are typically ER and PR negative whereas endometrial mucinous adenocarcinomas and endometrioid adenocarcinomas with mucinous differentiation are characteristically hormone receptor positive. Mesonephric adenocarcinomas are also characterized by negative ER and PR expression but may be distinguished from HPV-related and other non-HPV related cervical adenocarcinomas by their morphologic features and the expression of other markers such as GATA-3 (20,21). Endometrial endometrioid adenocarcinomas are also usually GATA-3 negative 20–24 but a greater proportion of high-grade tumors, including grade 3 endometrioid, serous and clear cell carcinomas, express this marker 25.

The morphologic and immunophenotypic features of true clear cell carcinomas at all sites in the female genital tract are identical. Thus, there are no markers that facilitate the distinction between clear cell carcinomas of the endometrium, cervix or ovary.

## DISTINCTION OF INDEPENDENT PRIMARY CARCINOMAS OF THE ENDOMETRIUM AND ADNEXA FROM METASTATIC SPREAD BETWEEN THESE SITES

Neoplasia involving the female genital tract can show multifocal anatomic distribution and this may present difficulty in determining whether individual tumors have arisen *in situ* (independent primary neoplasms), or whether one or more tumors represent metastases from another site 26. In the context of endometrial carcinoma, the most common dilemmas occur in the setting of (1) cases of synchronous endometrioid and ovarian endometrioid adenocarcinoma, and (2) concurrent endometrial and adnexal/peritoneal high-grade serous adenocarcinoma. With different morphologic subtypes of adenocarcinoma involving the endometrium and the ovary, it is usually relatively straightforward to determine that these represent synchronous independent primary neoplasms. Occasional diagnostic difficulties are also presented by nongynecologic tumors spreading to the endometrium, with or without adnexal involvement, and these will be addressed briefly.

One general principle used in determining tumor origin in pathology is the identification of a recognized tumor precursor such as *in situ* or intraepithelial carcinoma because the presence of such a lesion is considered strong evidence of tumor development at a particular anatomic site. However, it is worth noting that occasionally metastases can colonize preexisting mucosal structures and replace the native epithelium, thus closely mimicking *in situ* neoplasia. Such changes are well-described in the gynecologic tract 27–33; for example, endometrial serous adenocarcinoma may involve the mucosa of the fallopian tube and closely mimic serous tubal intraepithelial carcinoma (STIC). Therefore, some caution is warranted in the interpretation of both the presence and significance of apparent tumor precursors.

It has been estimated that 3% to 5% of patients with endometrial carcinoma also have ovarian carcinoma, while conversely 10% of ovarian carcinomas are associated with an endometrial carcinoma 26,34,35. Younger patients (<50 y) with endometrial carcinomas, and those with a family history of breast or ovarian cancer, have a higher risk of developing ovarian neoplasia 36. In a recent study of 327 women aged 15 to 49 years with atypical endometrial hyperplasia or carcinoma, 58%, 8%, and 34% of patients had hyperestrogenic factors, suspected Lynch syndrome, or neither risk factor, respectively 37. Synchronous ovarian cancer was present in 23% of patients with suspected Lynch syndrome and in 21% of patients without obvious risk factors but in only 7% of hyperestrogenic cases. Thus, synchronous endometrial and ovarian neoplasia appears to be as common in younger women without obvious endometrial cancer risk as in patients with possible Lynch syndrome 37.

### Synchronous Endometrial and Ovarian Endometrioid Adenocarcinoma

**FIG. 7:**
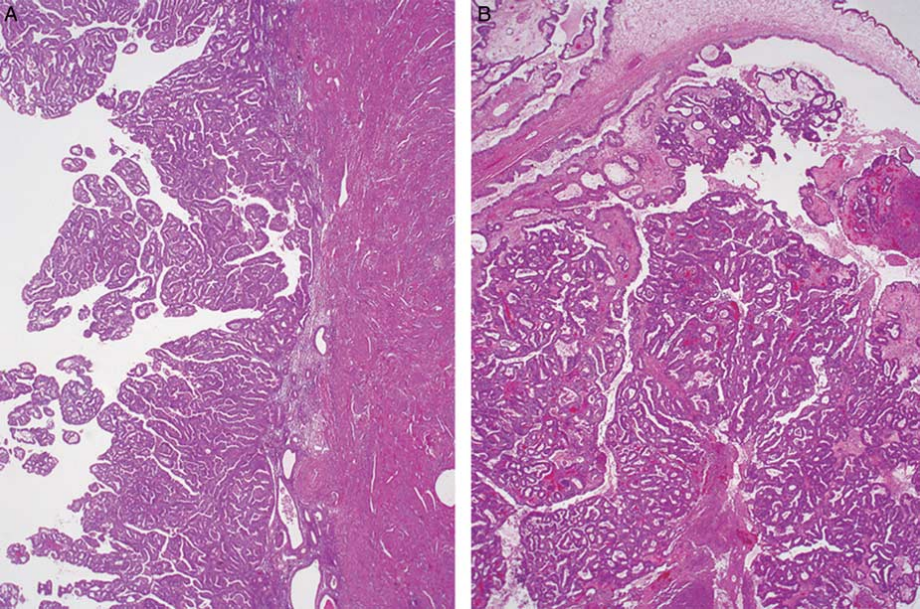
Synchronous, morphologically similar low-grade endometrioid adenocarcinomas of the endometrium (A) and ovary (B). The endometrial tumor is confined to the endometrium and the ovarian tumor (B) was associated with endometriosis (not shown). These tumors would be considered independent primary neoplasms using traditional clinicopathologic criteria.

This is a relatively common scenario and the tumors at both sites are usually low-grade carcinomas. Traditionally, the majority of such cases have been considered independent primary neoplasms, an interpretation supported by the generally favorable prognosis of these cases consistent with multifocal low-stage disease although there are few studies with long-term follow-up (Fig. 7) 38–49. However, as discussed below, this interpretation has been questioned by recent molecular studies. Synchronous independent tumors tend to occur in younger women than equivalent sporadic neoplasms but with conflicting data on the incidence in Lynch syndrome 20,50,51. The clinicopathologic features that aid in the distinction between apparent independent primary tumors and metastases in this clinical context are well-established and these are summarized in Table 3 52. Interpretative difficulties arise in those cases which show discordant features, some favoring primary independent neoplasms and others being more characteristic of metastasis (e.g. a low-grade endometrial adenocarcinoma with only superficial myometrial invasion associated with bilateral ovarian endometrioid adenocarcinomas in the absence of endometriosis). In most instances the endometrial carcinoma is recognizably a primary neoplasm, often supported by background features of atypical hyperplasia/endometrial intraepithelial neoplasia, and the difficulty lies with interpreting the nature of the ovarian tumor (primary vs. metastatic). However, the converse situation of tumor spread from the ovary to the endometrium does occur, and this may be suggested by the presence of a dominant tubo-ovarian tumor mass with limited, superficial and/or multifocal endometrial involvement 53. Similar interpretative difficulty may also be encountered in the less common situation where endometrial endometrioid adenocarcinoma is associated with an endometrioid adenocarcinoma of the fallopian tube with mucosal involvement 54. It should be noted that the criteria discussed above generally apply only to low-grade (FIGO grade 1 and grade 2) endometrioid adenocarcinomas as it is well established that high-grade endometrial carcinomas may present with metastases even when there is minimal myometrial invasion.

**TABLE 3:**
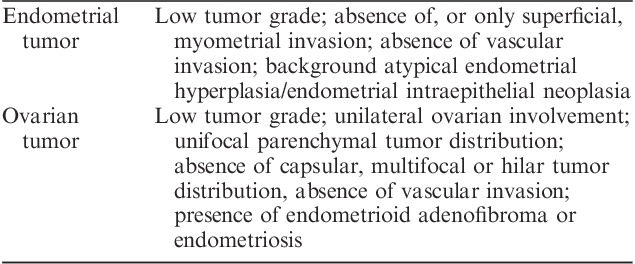
Traditional clinicopathologic features that favor an independent origin of synchronous endometrial and ovarian endometrioid carcinomas

Ancillary investigations have been used to aid diagnosis, particularly in cases where standard clinicopathologic criteria are ambiguous with regard to (usually ovarian) tumor origin. Immunohistochemistry has generally been considered of limited value as there is significant overlap in the immunoprofile of endometrial and ovarian endometrioid adenocarcinoma, not surprisingly given their morphologic similarity and the likely ultimate derivation of most ovarian endometrioid adenocarcinomas from the endometrium (with endometriosis as the intermediate step) 55. However, different beta-catenin and mismatch repair protein expression patterns can be helpful 50,56,57, and vimentin staining may also be useful as in one study 82% of endometrial endometrioid adenocarcinomas were positive, whereas 97% of ovarian endometrioid adenocarcinomas were negative 58. Molecular studies in this diagnostic context have included analysis of microsatellite instability, loss of heterozygosity, X-chromosome inactivation, the identification of *CTNNB1*, *KRAS*, and *PTEN* mutations, and novel techniques including mitochondrial DNA genotyping 20,55,59–66. Notably, in a significant number of cases there may be a discrepancy between interpretation of tumor origin based upon standard clinicopathologic features and molecular analysis, with the latter suggesting morphologic over-diagnosis of independent neoplasia 67. Recent studies using whole exome and high depth targeted massively parallel sequencing also suggest that most sporadic synchronous endometrial and ovarian endometrioid adenocarcinomas are clonally related and likely represent dissemination from one site to the other, probably mostly from the endometrium to the ovary 68–70. The apparent favorable prognosis of these cases may reflect transtubal migration and implantation of tumor cells (usually from the uterus to the ovary), contrasting with biologically aggressive (true) metastasis resulting from myometrial and vascular invasion.

In general, the interpretation of ancillary studies is based upon the premise that anatomically distinct tumors which share a significant number of immunohistochemical and/or molecular attributes are more likely to be clonal in origin (i.e. one primary neoplasm with metastasis in another organ), whereas those with different profiles are more likely to represent independent primaries. However, it is important to note that this may not always be the case 25. Multiple neoplasms could arise in different anatomical sites as a result of shared etiological factors (field effect), for example germline genetic predisposition or hormonal milieu, and these might be expected to demonstrate similar immunohistochemical and/or molecular alterations 71–74. Conversely, primary and metastatic lesions from a single tumor could differ due to tumor heterogeneity and progressive genetic alterations, particularly in metastatic sites. In practice, no single criterion is perfect and it is important to integrate all available clinicopathologic, immunohistochemical and molecular data in the assessment of problematic diagnostic cases.

### Synchronous Endometrial and Adnexal Serous Adenocarcinoma

Endometrial serous adenocarcinoma is an aggressive tumor that often presents at high-stage with extrauterine metastases. In those cases showing multiple adverse histologic factors such as deep myometrial, serosal, cervical and/or lymphovascular invasion, the extrauterine disease can reasonably be assumed to represent metastasis from the endometrium. This is also true even in cases of “minimal serous carcinomas,” some of which are confined to an endometrial polyp or show only “noninvasive” serous neoplasia (serous endometrial intraepithelial carcinoma, serous EIC), as it is well-recognized that such tumors can metastasize, possibly via transtubal dissemination 75–78.

**FIG. 8:**
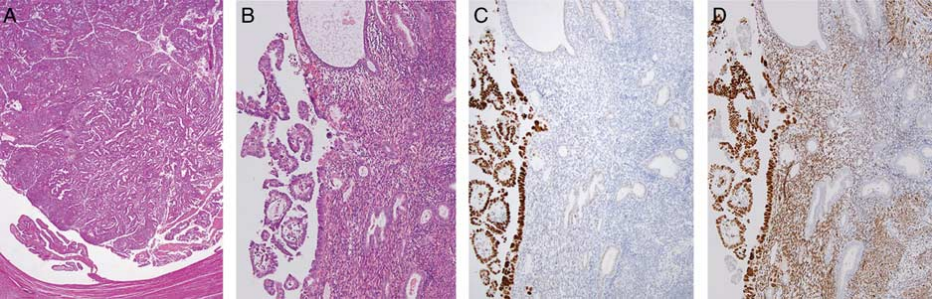
Fallopian tube high-grade serous adenocarcinoma distending the tubal lumen (A). Residual plical structures are present (lower left). Corresponding endometrium shows microscopic papillary tumor fragments and focal replacement of the surface epithelium by serous adenocarcinoma (B). The tumors at both sites show aberrant/mutation-type p53 staining (C) and WT1 expression (D) (endometrial tumor depicted) favoring the interpretation of a primary tubal carcinoma with spread to the endometrium.

**FIG. 9:**
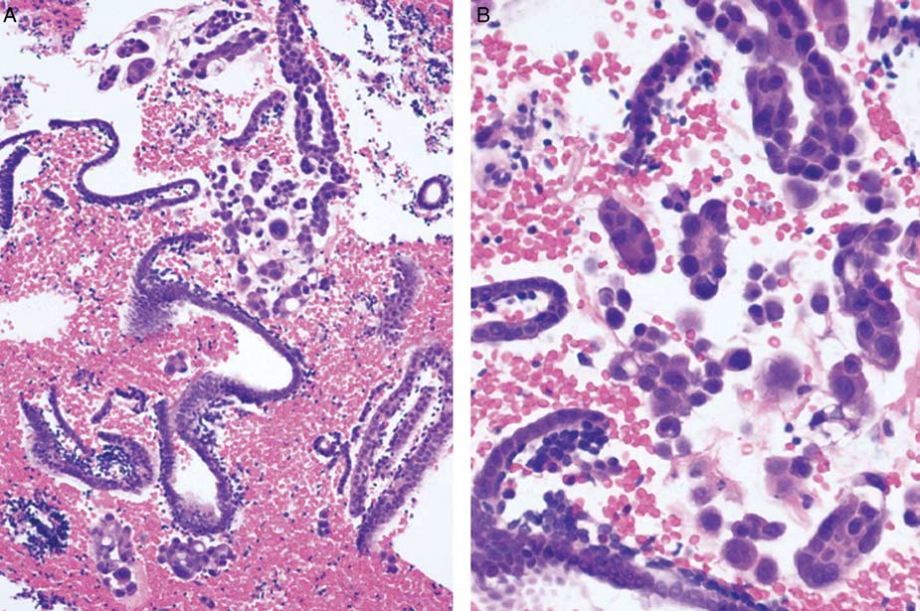
Endometrial biopsy showing “drop metastasis” from a primary tubal high-grade serous adenocarcinoma (A). The limited tumor material could be overlooked in an otherwise scant atrophic specimen. Higher magnification demonstrating the cytologic differences between the tumor and the inactive endometrium (B).

It is now widely accepted that most extrauterine pelvic high-grade serous adenocarcinomas arise from the fallopian tube, in particular the tubal fimbria where the precursor lesion STIC is most commonly identified 79. The presence of STIC is considered strong evidence for the tubal origin of such tumors and this is reflected in recent proposals regarding the assignation of the primary site in cases with high-grade serous neoplasia involving several extrauterine sites 80,81. However difficulty arises in cases where serous EIC or endometrial serous adenocarcinoma and STIC (with or without adnexal invasive serous adenocarcinoma) are both present (Fig. 8), and this finding is not uncommon when the tubes are thoroughly examined using the SEE-FIM (Sectioning and Extensive Examining of the Fimbriated End) protocol 82. For example, Jarboe et al. 76 identified adnexal neoplasia in 11/22 cases of endometrial serous adenocarcinoma; 6 patients had tubal involvement including 5 cases with STIC. Identical *TP53* mutations were demonstrated in two of the latter cases and the tumors at both sites showed similar p53 and WT1 immunostaining patterns. In a similar study of 55 endometrial serous adenocarcinomas where all tubal tissue was processed for histology, tubal neoplasia was present in 12 (22%) cases and 6 patients had extensive ovarian and/or omental disease 77. On the basis of the pathologic findings and WT1/p53 immunohistochemistry, 4 of these cases were favored to represent independent primary endometrial and tubal tumors, 3 primary adnexal malignancies with endometrial spread/implantation, and 3 primary endometrial tumors with direct spread to the tubal isthmus; in 2 cases tumor origin remained uncertain. Intraluminal tumor cells were identified in 10/12 cases with concurrent endometrial and tubal neoplasia suggesting transluminal spread as a mechanism for tumor dissemination, whether antegrade or retrograde. Jia et al. 83 investigated 21 patients with serous EIC and extrauterine high-grade serous adenocarcinoma. On the basis of *TP53* mutation analysis, 10 cases were interpreted to represent disseminated endometrial serous adenocarcinoma, 5 cases were favored to be primary adnexal/tubal tumors, and 6 cases showed mixed (endometrial and adnexal) origin of the metastatic deposits; “free floating tumor cells” (intraluminal tumor cells) were identified in 5 of the cases. In a study by Tolcher et al. 84, tubal involvement was present in 11/38 patients with endometrial serous adenocarcinoma. While most cases appeared to represent tubal metastasis from the endometrial tumor, 3 demonstrated STIC suggesting a tubal primary or synchronous endometrial and tubal neoplasia in a minority of cases. Mingels et al. 85 found that complete sectioning of the endometrium revealed serous EIC in 15% of patients with apparent primary pelvic high-grade serous carcinomas. Recently, Kommoss and colleagues reported tubal involvement in 32/161 (20%) cases of endometrial serous adenocarcinoma (including cases of carcinosarcoma with a component of serous adenocarcinoma, and carcinomas with mixed differentiation) 32. Seventeen of these cases showed STIC-like features, but in 14 of these the overall histologic and immunohistologic features were favored to represent a primary endometrial serous adenocarcinoma with tubal metastasis. Overall, these studies demonstrate the difficulty in assigning tumor origin when there is multifocal high-grade serous neoplasia, and that the presence of an apparent *in situ* lesion (such as STIC) does not necessarily equate to tumor origin at a specific site. From a practical perspective, the presence of scant cells from a high-grade serous adenocarcinoma in an endometrial biopsy specimen should raise the possibility of “drop metastasis” from a tumor of adnexal or peritoneal origin (Fig. 9) 86.

As regards immunohistochemistry in this diagnostic setting, WT1 immunoreactivity in a high-grade serous carcinoma, particularly if diffuse, favors tubo-ovarian origin (90%–95% of tubo-ovarian high-grade serous adenocarcinomas are WT1 positive) (Fig. 8). However, a significant proportion of endometrial serous adenocarcinomas can show WT1 immunoreactivity with 44% of cases in one study exhibiting some degree of positivity 87. In contrast, HER2 overexpression appears more characteristic of endometrial serous adenocarcinoma although a wide range in the proportion of positive cases has been reported 88–91. Mutation-type p53 staining is almost universal in endometrial and tubo-ovarian high-grade serous adenocarcinomas but different expression patterns (e.g. null vs. overexpression) in different anatomic sites would suggest independent neoplasms. We make the point that in most cases of high-grade serous neoplasia at more than one site this will represent a primary at one site with metastasis to the other.

In summary:

1. Endometrial and adnexal high-grade serous carcinomas may arise independently but most cases represent spread from the endometrium to adnexal/peritoneal sites, and others secondary involvement of the endometrium (drop metastasis) from a likely tubal primary.
2. Intraluminal tubal dissemination may account for tumor spread in some cases, particularly those showing minimal/absent stromal invasion.
3. Difficulty in assigning tumor origin persists in a minority of cases, even following exhaustive histologic sampling, immunohistochemistry and molecular analysis.

## NONGYNECOLOGIC METASTASIS TO THE ENDOMETRIUM

**FIG. 10:**
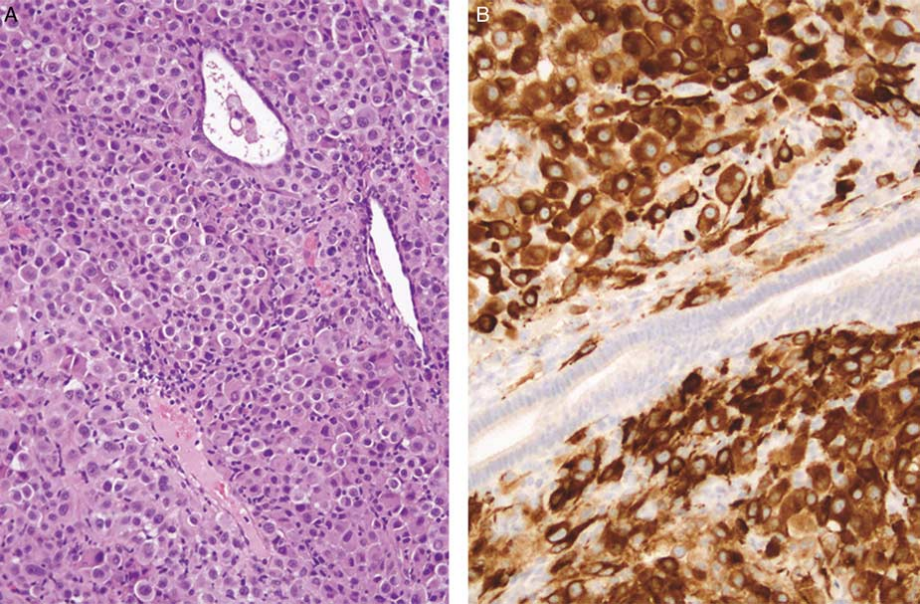
Malignant melanoma metastatic to the endometrium (A). Only focal residual benign endometrial glands are present and the tumor infiltrates around these. The tumor cells express melan-A whereas a normal endometrial gland (center) is not stained (B).

**FIG. 11:**
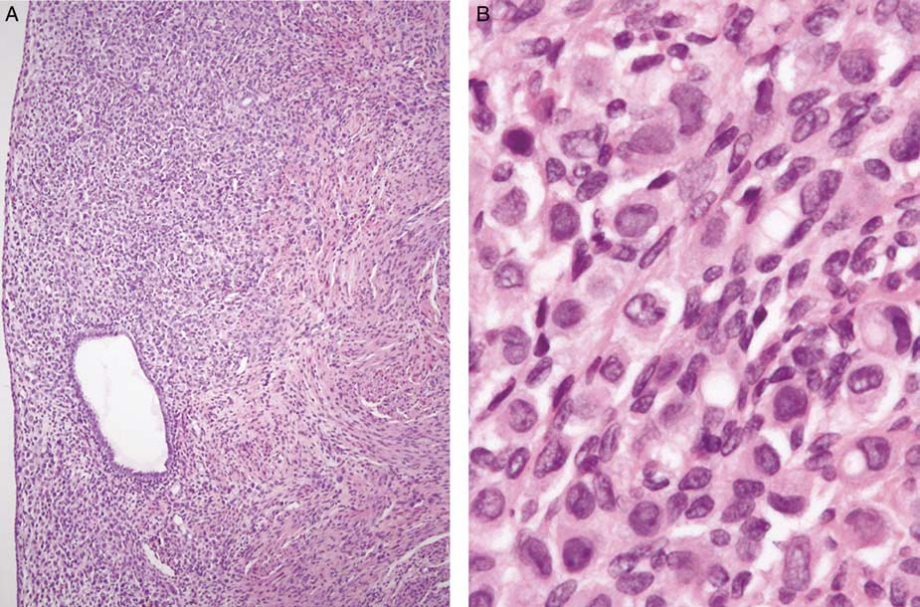
Lobular carcinoma of breast metastatic to the endometrium infiltrating around normal endometrial glands (A). The changes are relatively subtle but the malignant nature of the infiltrate is apparent at higher magnification (B).

Nongynecologic tumors occasionally spread to the uterus and may be identified in endometrial biopsy or hysterectomy specimens. Isolated uterine metastases are most commonly derived from the breast, colon, and stomach but virtually any malignant tumor can potentially involve the endometrium 53,92. Clinical history is clearly important in this situation as often the patient will have an established diagnosis of previous malignancy at another site. However, this is not always the case and the relevant information may not be provided to the pathologist. In many cases, the morphologic differences between the metastasis and the usual types of primary endometrial neoplasia will prompt consideration of extrauterine tumor origin, and the instigation of appropriate immunohistochemical analysis as required (Fig. 10). However, potential diagnostic problems can arise when metastatic involvement is subtle (Fig. 11), or where there is histologic overlap between primary endometrial and extrauterine tumors, for example tumors with a papillary growth pattern 93. Morphologically poorly differentiated or undifferentiated malignancies of epithelial or nonepithelial type can also cause difficulty, but in the majority of cases the diagnosis can be resolved with clinical correlation and appropriate immunohistochemistry 53,92. Clues to the metastatic nature of a tumor in the endometrium may be multifocal involvement, lack of an identifiable precursor lesion, and a pattern of infiltration which surrounds rather than destroys normal glands (Fig. 11) 94.

## RECOMENDATIONS

1. It is of considerable importance to distinguish between an endometrial and cervical origin for a carcinoma given the marked differences in management.
2. In the distinction between an endometrial and a cervical origin for an adenocarcinoma, the panels of markers which are useful will depend on the morphologic subtype and not just the site of origin.
3. In the distinction between a high-risk HPV-related (usual type) endocervical adenocarcinoma and a low-grade endometrial endometrioid adenocarcinoma, the most useful immunohistochemical markers are p16 and hormone receptors (ER and PR); however, it is stressed that there may be unexpected positive and negative staining reactions with any of the markers. HPV studies will be of value in such cases.
4. In the distinction between a high-risk HPV-related (usual type) endocervical adenocarcinoma and a high-grade endometrial adenocarcinoma, p53 immunohistochemistry and HPV studies may be of value. Most endometrial serous adenocarcinomas and some other high-grade endometrial carcinomas exhibit mutation-type p53 staining and are always HPV negative. High-risk HPV-related endocervical adenocarcinomas rarely, if ever, exhibit mutation-type p53 expression.
5. Different panels of markers will be useful in the distinction between a non–HPV-related cervical adenocarcinoma and an endometrial adenocarcinoma.
6. Multifocal tumor distribution is relatively common in carcinomas arising in the female genital tract. Accurate designation of tumor origin is not always straightforward but is important for staging, prognosis and management. Histologic, immunophenotypic and/or molecular findings should be integrated as far as possible, particularly in diagnostically problematic cases.
7. Recent molecular studies suggest that the traditional view that most synchronous low-grade endometrial and ovarian endometrioid adenocarcinomas are independent neoplasms may be incorrect. The preliminary findings have to be validated in larger studies but if confirmed there may be a future requirement to modify current staging parameters given the apparent good prognosis of this group of patients.
8. While rare, the possibility of nongynecologic origin should be considered in uterine neoplasms with unusual histologic features, with appropriate clinical correlation and immunohistochemical analysis.
